# Studies on Antibacterial Activity and Diversity of Cultivable Actinobacteria Isolated from Mangrove Soil in Futian and Maoweihai of China

**DOI:** 10.1155/2019/3476567

**Published:** 2019-06-09

**Authors:** Feina Li, Shaowei Liu, Qinpei Lu, Hongyun Zheng, Ilya A. Osterman, Dmitry A. Lukyanov, Petr V. Sergiev, Olga A. Dontsova, Shuangshuang Liu, Jingjing Ye, Dalin Huang, Chenghang Sun

**Affiliations:** ^1^Institute of Medicinal Biotechnology, Chinese Academy of Medical Sciences & Peking Union Medical College, Beijing 100050, China; ^2^College of Basic Medical Sciences, Guilin Medical University, Guilin 541004, China; ^3^Center of Life Sciences, Skolkovo Institute of Science and Technology, Moscow 143025, Russia; ^4^Department of Chemistry, A.N. Belozersky Institute of Physico-Chemical Biology, Lomonosov Moscow State University, Moscow 119992, Russia; ^5^Shemyakin-Ovchinnikov Institute of Bioorganic Chemistry, The Russian Academy of Sciences, Moscow 117997, Russia; ^6^China Pharmaceutical University, Nanjing 210009, China

## Abstract

Mangrove is a rich and underexploited ecosystem with great microbial diversity for discovery of novel and chemically diverse antimicrobial compounds. The goal of the study was to explore the pharmaceutical actinobacterial resources from mangrove soil and gain insight into the diversity and novelty of cultivable actinobacteria. Consequently, 10 mangrove soil samples were collected from Futian and Maoweihai of China, and the culture-dependent method was employed to obtain actinobacteria. A total of 539 cultivable actinobacteria were isolated and distributed in 39 genera affiliated to 18 families of 8 orders by comparison analysis of partial 16S rRNA gene sequences. The dominant genus was* Streptomyces* (16.0 %), followed by* Microbacterium *(14.5 %),* Agromyces *(14.3 %), and* Rhodococcus* (11.9 %). Other 35 rare actinobacterial genera accounted for minor proportions. Notably, 11 strains showed relatively low 16S rRNA gene sequence similarities (< 98.65 %) with validly described species. Based on genotypic analyses and phenotypic characteristics, 115 out of the 539 actinobacterial strains were chosen as representative strains to test their antibacterial activities against “ESKAPE” bacteria by agar well diffusion method and antibacterial mechanism by the double fluorescent protein reporter system. Fifty-four strains in 23 genera, including 2 potential new species, displayed antagonistic activity in antibacterial assay. Meanwhile, 5 strains in 3 genera exhibited inhibitory activity on protein biosynthesis due to ribosome stalling. These results demonstrate that cultivable actinobacteria from mangrove soil are potentially rich sources for discovery of new antibacterial metabolites and new actinobacterial taxa.

## 1. Introduction

Currently, antibiotic resistance is occurring more and more severely and already has become a global challenge to public health [1, 2]; however, new types of antibacterial drugs are so extremely limited that clinicians are forced to the situation as “Bad bugs, No drugs.” In early 2017, a request was made to the World Health Organization (WHO) by member states to develop a global priority pathogen list (PPL) of antibiotic-resistant bacteria to help in prioritising the research and development of new and effective antibiotic treatments [3, 4].

Actinobacteria, especially, the genus* Streptomyces*, are major producers of bioactive secondary metabolites [5, 6]. After decades of screening, it has become increasingly difficult to discover new antibiotics from actinobacteria isolated from common soil environments. Nowadays, more and more researches are focused on special habitats and extreme environments [7, 8], such as desert [9], marine [10], and mangrove [11], since microbes in special environments have to develop unique defense mechanism against the stress from their habitats and can evolve adaptive biosynthetic pathways for synthesizing novel biological compounds [12]. In fact, a large number of new bioactive compounds produced by actinobacterial strains residing in special environments have been discovered in recent years [13–15].

Mangrove is unique intertidal ecosystem with the condition of high moisture, high salinity, low oxygen, and high organic matter content [16, 17]. Because the mangrove soil conditions are extremely different from common terrestrial conditions, microorganisms especially actinobacteria in mangrove soil have distinctive adaptation characteristics and have the potential to produce novel bioactive metabolites [18]. Investigations in many countries indicated that the mangrove actinobacteria have rich diversity and various biological activities [6, 13, 16, 19, 20]. At the time of writing, at least 86 new actinobacterial species including 8 novel genera have been isolated from mangrove. In addition, more than 84 new compounds produced by mangrove actinobacteria including some attractive structures such as salinosporamides, xiamycins, and novel indolocarbazoles [21, 22] have been reported. From north to south, mangroves in China mainly distribute along the southeast coast including Zhejiang province, Fujian province, Guangdong province, and Guangxi Zhuang Autonomous Region. Among them, Guangdong and Guangxi possess most of the mangrove area [23, 24].

In order to explore the antibacterial resources and gain insight into the diversity of cultivable actinobacteria, mangrove soil samples from Futian, Guangdong, and Maoweihai, Guangxi, were collected and investigated. Due to the high prevalence of multidrug resistance among “ESKAPE” bacteria, defined by the Infectious Diseases Society of America as* Enterococcus faecium*,* Staphylococcus aureus*,* Klebsiella pneumoniae*,* Acinetobacter baumannii*,* Pseudomonas aeruginosa,* and* Enterobacter* spp., these pathogens in the global PPL of antibiotic-resistant bacteria were selected as the indicator bacteria in this study. In addition, a high-efficiency pDualrep2 reporter system was combined to accelerate the discovery of actinobacterial strains with clearly antibacterial mechanism from mangrove soil.

## 2. Materials and Methods

### 2.1. Collection of Mangrove Soil Sample

A total of ten soil samples were collected from 2 mangrove reserves of China in August, 2017. Two samples were collected from Futian, Shenzhen, Guangdong province and 8 from Maoweihai, Qinzhou, Guangxi Zhuang Autonomous Region. The information for the samples is listed in Table 1. All the samples were packed in sterilized envelopes and brought to the laboratory at the earliest possible time. Prior to grinding with mortar and pestle, each sample was immediately air-dried in the laminar flow hood at room temperature for 2 days.

### 2.2. Cultivable Actinobacteria Isolation and Maintenance

Ten media were prepared to isolate the actinobacterial strains (Table S1). All the isolation media were added 3 % seawater. In addition, nalidixic acid (20 mg/L), cycloheximide (50 mg/L), and potassium dichromate (50 mg/L) were also added in the media to prevent the growth of Gram-negative bacteria and fungi.

Actinobacteria were isolated by using dilution plating technique as described by Li et al. [25]. 0.2 mL of 10^−2^ soil suspension was spread onto isolation agar plates. After incubation at 28°C for 2-4 weeks, colonies were picked up and streaked on the freshly prepared YIM 38 medium (1 L sterile water: 4.0 g glucose, 4.0 g yeast extract powder, 5.0 g malt extract powder, 15.0 g agar, pH 6.0) to obtain the pure isolates. The pure cultures were maintained on YIM 38 agar slants at 4°C for several weeks and also preserved in glycerol suspensions (20 %, v/v) at −80°C.

### 2.3. PCR Amplification and Sequencing of 16S rRNA Gene

Genomic DNA was extracted as described by Li et al. [26] and used as the template to amplify the 16S rRNA gene by PCR with the primers 27F (5′-AGAGTTTGATCCTGGCTCAG-3′) and 1492R (5′-GGTTACCTTGTTACGACTT-3′) [27]. The reaction mixture (50 *μ*L) contained 25 *μ*L 2×supermix (TransGen, Beijing), 1 *μ*L each of the primers (10 mM, Sangon Biotech, Beijing), 1.5 *μ*L DNA, and 21.5 *μ*L ddH_2_O. The PCR amplification included the following parameters: (i) 95°C for 3 min (initial denaturation), (ii) 30 cycles of 94°C for 1 min (denaturation), 60°C for 1 min (annealing), and 72°C for 1 min (extension), and (iii) 72°C for 10 min (final extension). The amplicons were then visualized by gel electrophoresis using 5 *μ*L of PCR product in a 1 % agarose gel. The PCR products were purified and then sequenced on the ABI PRISM™ 3730XL DNA Analyzer (Foster City, CA).

### 2.4. Sequence Analysis

The 16S rRNA gene sequences obtained were compared with those of the type strains available in NCBI (http://www.ncbi.nlm.nih.gov/) and the EzBioCloud (https://www.ezbiocloud.net/) [28] using the Basic Local Alignment Search Tool (BLAST) [29] to determine an approximate phylogenetic affiliation of each strain. The corresponding sequences of closely related type species were retrieved from GenBank database using the EzBioCloud server. Multiple alignments were made using CLUSTAL_X tool in MEGA version 7.0 [30]. Phylogenetic tree based on neighbour-joining method [31] was constructed using the MEGA version 7.0. Evolutionary distances were calculated using the Kimura's two-parameter model [32]. The topology of the phylogenetic tree was evaluated by bootstrap method with 1000 replications [33].

### 2.5. Nucleotide Sequence Accession Numbers

The sequences obtained in this study were deposited in GenBank with the 16S rRNA gene sequences under the accession numbers: MK589722 -MK589799 and MK685120.

### 2.6. Small-Scale Fermentation

To check the antibacterial potential of isolated actinobacterial strains, small-scale fermentation was performed. One hundred and fifteen representative strains were selected based on analyses of partial 16S rRNA gene sequences and phenotypic characteristics. Each strain was inoculated separately in six of 500 ml Erlenmeyer flasks containing 100 ml of YIM 38 broth medium. After being incubated for 7 days at 28°C with shaking (at 180 rpm), the 600 ml fermentation broth was centrifuged and its supernatant was extracted twice with ethyl acetate (EtOAc, 1:1, v/v). Organic layer was dried up by rotary evaporation, and residue was dissolved in 3 ml of methanol. Sixty milliliter of water layer was lyophilized, and then its residue was dissolved in 3 ml of 50 % methanol-water. The mycelium was soaked overnight in acetone and then filtered. The acetone extract was dried in vacuo and dissolved in 3 ml of 50 % methanol-water. Ultimately, each strain has three kinds of sample for antibacterial assay.

### 2.7. Antibacterial Screening

Six sets of indicator bacteria were used in antibacterial assay. Each set consisted of two strains, one was sensitive strain and another was drug-resistant strain. The indicator bacteria were* Enterococcus faecalis *(*E*.* faecalis*, ATCC 33186, 310682),* Staphylococcus aureus *(*S*.* aureus*, ATCC 29213, ATCC 33591),* Klebsiella pneumoniae *(*K*.* pneumoniae*, ATCC 10031, ATCC 700603),* Acinetobacter baumannii *(*A*.* baumannii*, 2799, ATCC 19606),* Pseudomonas aeruginosa *(*P*.* aeruginosa*, ATCC 27853, 2774), and* Escherichia coli *(*E*.* coli*, ATCC 25922, ATCC 35218).* E*.* faecalis* 310682,* A*.* baumannii* ATCC 19606, and* E*.* coli* ATCC 35218 are resistant to vancomycin, carbapenems, and ampicillin, respectively.* S*.* aureus* ATCC 33591 is resistant to both cefoxitin and oxacillin. Both* K*.* pneumoniae* ATCC 700603 and* P*.* aeruginosa* 2774 are resistant to aminoglycosides; meanwhile,* K*.* pneumoniae* ATCC 700603 is resistant to *β*-lactam antibiotics and* P*.* aeruginosa* 2774 is resistant to carbapenems. Indicator bacteria were obtained either from American Type Culture Collection (ATCC) or from the clinic and deposited in Institute of Medicinal Biotechnology, Chinese Academy of Medical Sciences.

Antibacterial assay was performed using agar well diffusion method [34]. After drying up, paper disk (diameter 6 mm with 60 *μ*L prepared sample) was placed on Mueller-Hinton (MH) agar containing the indicator bacteria. Meanwhile, 60 *μ*L methanol without sample and with 1 *μ*g levofloxacin was used as the negative control and positive control, respectively. The plates were incubated at 37°C for 24 h, and the antibacterial activity was evaluated by measuring the inhibition zone.

### 2.8. Mechanism of Action Determination

Ribosome and DNA biosynthesis inhibitors were screened by the double fluorescent protein reporter system with reporter strain JW5503-pDualrep2 [35]. Briefly, 100 *μ*L of ethyl acetate extract was dried up in laboratory hood and 100 *μ*L DMSO was added as sample to be tested. 2 *μ*L of sample was applied to agar plate containing a lawn of the reporter strain. After overnight incubation at 37°C, the plate was scanned by ChemiDoc (Bio-Rad) system with two channels including “Cy3-blot” (553/574 nm, green pseudocolor) for RFP fluorescence and “Cy5-blot” (588/633 nm, red pseudocolor) for Katushka2S fluorescence. Induction of Katushka2S expression is triggered by translation inhibitors, while RFP is upregulated by induction of DNA damage SOS response. Levofloxacin and erythromycin were used as positive controls for DNA biosynthesis and ribosome inhibitors, respectively.

## 3. Result

### 3.1. Isolation and Diversity of Cultivable Actinobacteria from Mangrove Soil

**Figure 1:**
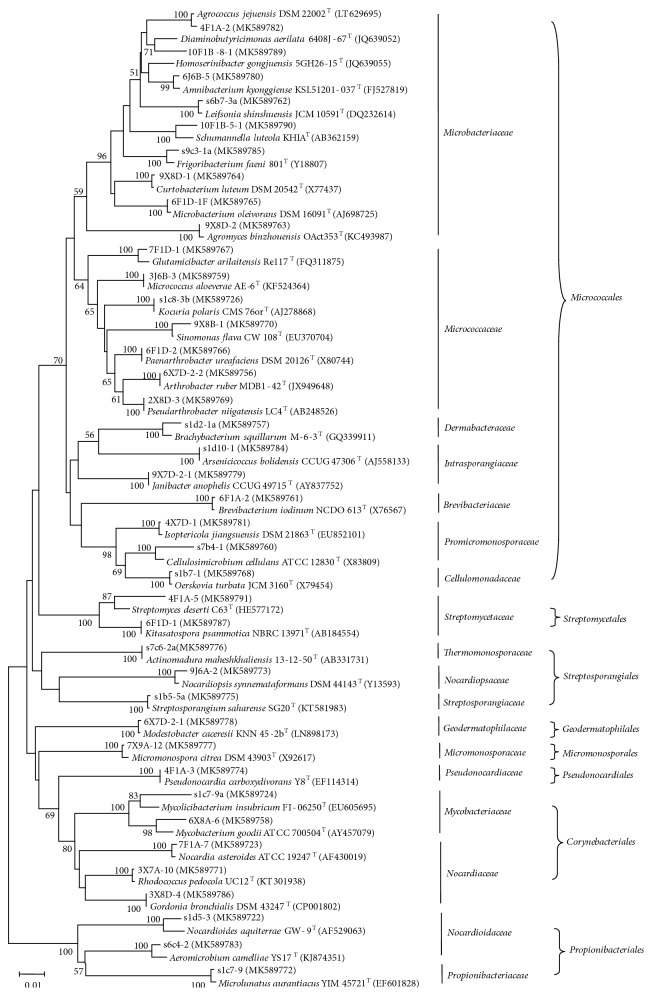
Phylogenetic tree based on the 16S rRNA gene sequences using neighbour-joining method for the representative actinobacterial strains and their closely related type strains. Numbers at nodes indicate the level of bootstrap support based on 1000 replications (only values > 50 % are shown). Bar, 1 nt substitutions per 100 nt.

Among 843 isolates obtained, 539 isolates were identified as actinobacterial strains by partial 16S rRNA gene sequence comparison analysis and further assigned to 39 genera in 18 families of 8 orders as follows:* Streptomyces*,* Microbacterium*,* Agromyces*,* Rhodococcus*,* Sinomonas*,* Mycobacterium*,* Curtobacterium*,* Arthrobacter*,* Nocardia*,* Kocuria*,* Paenarthrobacter*,* Nocardiopsis*,* Glutamicibacter*,* Brachybacterium*,* Agrococcus*,* Isoptericola*,* Aeromicrobium*,* Kitasatospora*,* Mycolicibacterium*,* Micrococcus*,* Arsenicicoccus*,* Brevibacterium*,* Schumannella*,* Leifsonia*,* Cellulosimicrobium*,* Gordonia*,* Micromonospora*,* Homoserinibacter*,* Pseudarthrobacter*,* Amnibacterium*,* Frigoribacterium*,* Oerskovia*,* Janibacter*,* Streptosporangium*,* Actinomadura*,* Modestobacter*,* Pseudonocardia*,* Nocardioides*, and* Microlunatus* (Figure 1). The predominant genus was* Streptomyces* (16.0 %, 86 strains), followed by* Microbacterium *(14.5 %, 78 strains),* Agromyces *(14.3 %, 77 strains), and* Rhodococcus* (11.9 %, 64 strains) (Table 2).

**Figure 2:**
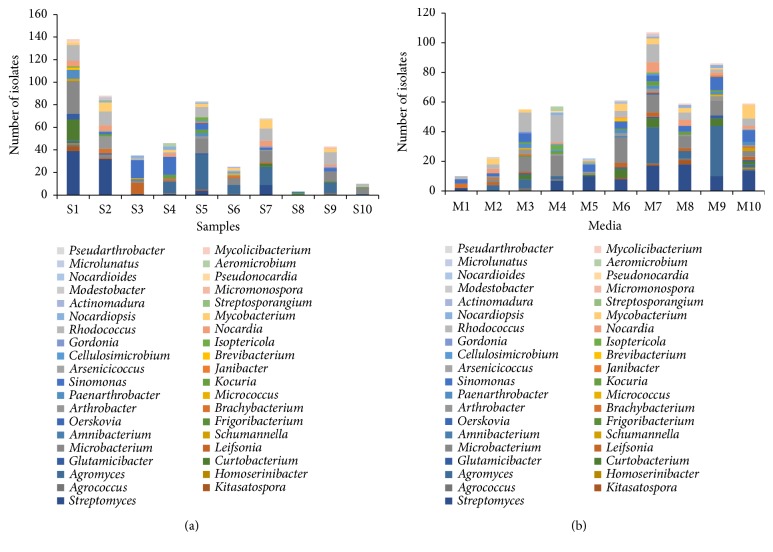
Diversity of cultivable actinobacteria from mangrove soil in Futian and Maoweihai. (a) Number of actinobacterial isolates from different samples. (b) Number of actinobacterial isolates recovered from the different culture media.

The distribution of the 539 actinobacterial strains from 10 samples is displayed in Figure 2(a) and Table S2. Sample 2 gave the highest diversity (18 genera), followed closely by sample 1 (17 genera), sample 5 (16 genera), sample 4 (13 genera), sample 7 (12 genera), both sample 6 and sample 9 (11 genera), sample 3 (8 genera), sample 10 (4 genera), and sample 8 (2 genera). Among the 10 different media used for isolation of actinobacteria, M7 generated the most successful isolation according to the number and diversity of obtained isolates as shown in Figure 2(b) and Table S3. Totally, 107 actionbacterial strains distributed in 23 genera were obtained from M7. M10 produced the second-highest diversity of isolates (18 genera), and M9 generated the second-highest number of isolates (86 strains). Meanwhile, M1 yielded the lowest number and diversity of isolates (10 strains in 4 genera).

### 3.2. Novelty of Cultivable Actinobacteria

**Figure 3:**
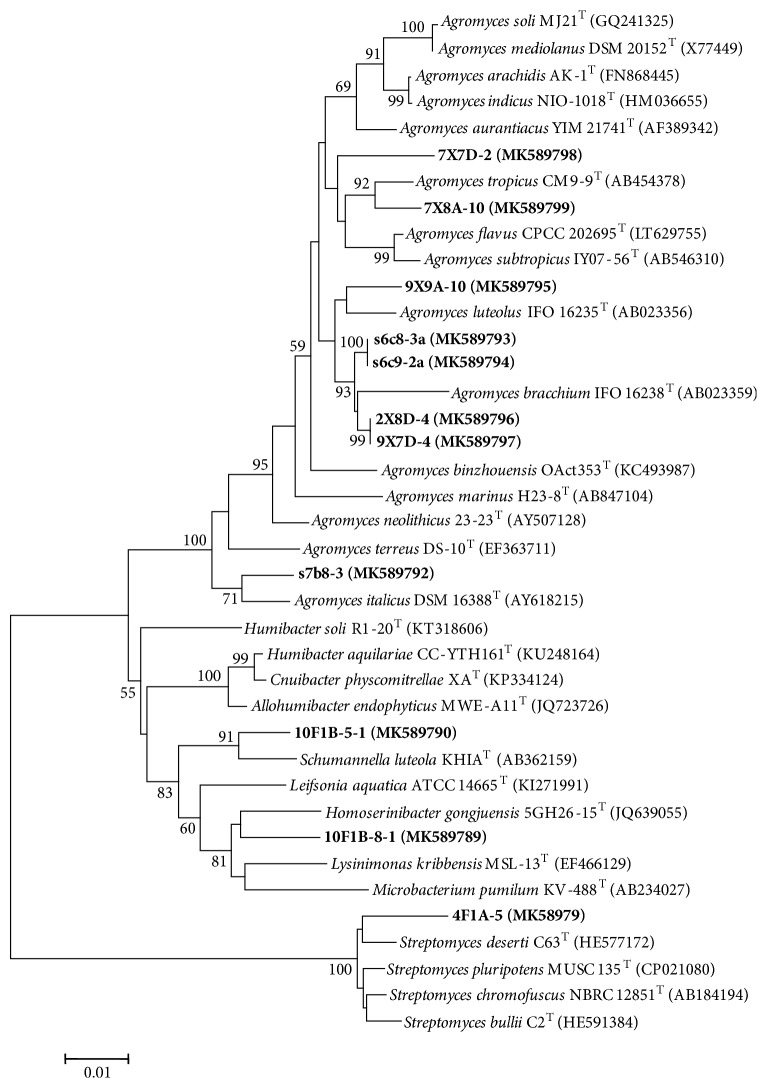
Neighbor-joining phylogenetic tree based on almost full-length 16S rRNA gene sequences of 11 potential novel strains and their closely related type strains. Numbers at nodes indicate the level of bootstrap support based on 1000 replications (only values > 50 % are shown). Bar, 1 nt substitutions per 100 nt.

Among the 539 actinobacterial strains, 11 strains exhibited low 16S rRNA gene sequence similarities (< 98.65 %, the threshold for differentiating two species) [36] with validly described species based on the results of BLAST search in EzBiocloud (Table 3), which indicated that these isolates could represent novel taxa. The phylogenetic tree based on almost full-length 16S rRNA gene sequences (Figure 3) showed these potential novel strains were assigned to 4 genera including* Agromyces* (8 strains),* Homoserinibacter* (1 strain),* Schumannella* (1 strain), and* Streptomyces* (1 strain). These strains will be further identified with a polyphasic approach to determine their taxonomic positions.

### 3.3. Antibacterial Activity of Actinobacterial Isolates

**Figure 4:**
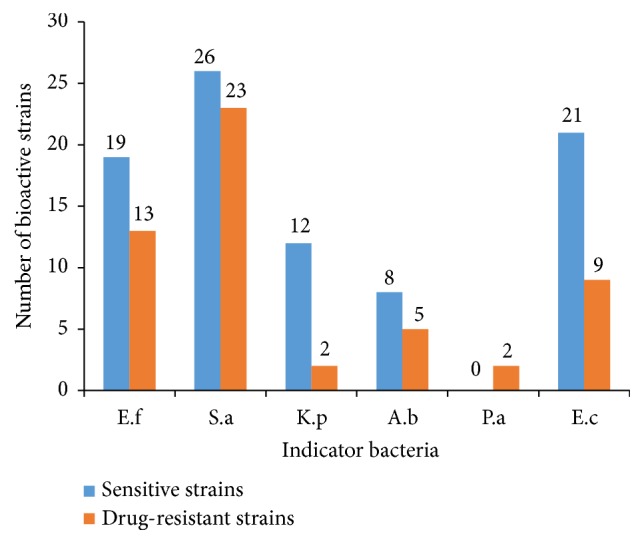
The antibacterial profiles of the actinobacteria against “ESKAPE” bacteria (E.f:* Enterococcus faecalis*; S.a:* Staphylococcus aureus*; K.p:* Klebsiella pneumoniae*; A.b:* Acinetobacter baumannii*; P.a:* Pseudomonas aeruginosa*; E.c:* Escherichia coli*).

Among the 115 strains selected for antibacterial assay, 54 strains, affiliated to 23 different genera, showed antagonistic activity against at least one of the indicator bacteria (Table 2). The antibacterial profiles of the 54 strains against “ESKAPE” bacteria are shown in Figure 4. Among them, 37 strains were active against at least one of Gram-positive bacteria and 32 strains were active against at least one of Gram-negative bacteria; meanwhile, 16 strains exhibited antibacterial activity against both Gram-positive and Gram-negative bacteria.

### 3.4. Mechanism of Action Determination

**Figure 5:**
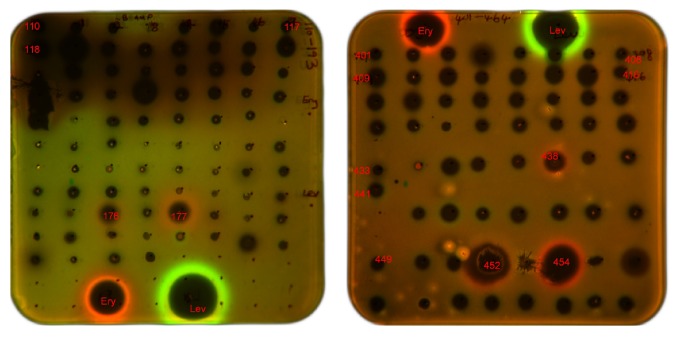
Induction of a two-color dual reporter system sensitive to inhibitors of the ribosome progression or inhibitors of DNA replication, respectively. Spots of erythromycin (Ery), levofloxacin (Lev), and tested samples were placed on the surface of an agar plate containing* E*.* coli* tolC cells transformed with the pDualrep2 reporter plasmid. Shown is the fluorescence of the lawn of* E*.* coli* cells scanned at 553/574 nm (green pseudocolor) for RFP fluorescence and 588/633 nm (red pseudocolor) for Katushka2S fluorescence. Induction of Katushka2S expression is triggered by translation inhibitors, while RFP is upregulated by induction of DNA damage SOS response. 176: 10X7D-1-3; 177: 7X8A-5; 438: s1d5-4; 452: s7b4-1; 454: s1b9-3.

Ethyl acetate extracts from the culture broths of 115 strains were screened by the double fluorescent protein reporter system. Five strains, including 3 strains (strains 10X7D-1-3, 7X8A-5, and s1b9-3) in genus* Streptomyces*, 1 strain (strain s1d5-4) in genus* Micromonospora*, and 1 strain (strain s7b4-1) in genus* Cellulosimicrobium*, induced Katushka2S expression as erythromycin did. Meanwhile, no strain induced SOS-response as levofloxacin did (Figure 5).

## 4. Discussion

Actinobacteria are widely dispersed throughout the mangrove environments [21, 37]. Previous studies exhibited 34 actinobacterial genera have ever been isolated from mangrove soil in Futian and Maoweihai [38–47]. In this study, 226 actinobacteria in 29 genera and 313 actinobacteria in 31 genera were isolated from samples collected from Futian and Maoweihai, respectively. Twenty-one genera recovered were shared by both Futian and Maoweihai. The combination of 10 culture media and 10 mangrove soil samples led to the discovery of 39 actinobacterial genera and 11 potential new species, which not only provided more diverse strains for assay, but also demonstrated that it is necessary to use various types of isolation media to increase in the number and diversity of actinobacteria. Mangrove microorganisms especially actinobacteria have been reported to have the ability to produce structurally unique and bioactive natural products [16, 21, 48]. According to the report of Xu et al. [21], about 73 novel compounds have been reported from mangroves originated actinobacteria and among these, 40 new compounds were reported from actinobacteria isolated from the mangrove soil samples only, which shows that actinobacteria from mangrove soil have great advantage to produce new bioactive metabolites.

In the antibacterial assay, 54 strains affiliated to 23 genera, including 26 strains in 11 genera from Futian samples and 28 strains in 15 genera from Maoweihai samples, exhibited inhibitory activities against at least one of “ESKAPE” bacteria as shown in Table S4. These active strains consisted of 20 strains in genus* Streptomyces* and 34 strains in 22 rare genera. The predominant active strains belong to genus* Streptomyces*, which is in line with the previous reports [19, 39, 49]. Twenty* Streptomycete* strains, including a potential new species designated as strain 4F1A-5, showed inhibitory activity against at least one of Gram-positive bacteria, and 8 of them also showed activity against at least one of Gram-negative bacteria. As the biggest genus in actinobacteria,* Streptomyces* contains 848 species and 38 subspecies (http://www.bacterio.net/streptomyces.html), members of genus* Streptomyces* are well-known as the main sources of antibiotics with diverse biological activities and chemical structures [50], since they usually harbor the large genome size and possess a number of biosynthetic gene clusters that encode multifunctional biosynthetic enzymes [51, 52].

Rare actinobacteria also are important sources in the discovery of novel antibiotics [53]. Recently, mangromicins, a group of new secondary metabolites with unique chemical structures, were found from* Lechevalieria aerocolonigenes* K10-0216 isolated from a mangrove sediment sample by Omura's group [54–56], which further indicated the rare actinobacteria deserve to be studied extensively to find new antibiotics. In the present study, several active strains in 22 rare genera such as* Sinomonas*,* Pseudarthrobacter*,* Leifsonia,* and* Gordonia* have been rarely studied. Notably, strain 10F1B-8-1, as a potential new species in rare genus* Homoserinibacter*, showed broad-spectrum antibacterial activity (Table S4) and is definitely worth studying in priority.

pDualrep2 reporter system is a very sensitive screening model for sorting out antibiotic's mechanisms of action, which can distinguish simultaneously between antibiotics that induce the SOS response due to DNA damage and cause the Katushka2S expression due to ribosome stalling. The existence of ribosome inhibitors such as erythromycin will lead Katushka2S expression, and the existence of inhibitors of DNA biosynthesis such as levofloxacin will lead RFP expression. In this study, screening results indicated 5 strains produced inhibitors of ribosome, but none produced inhibitors of DNA biosynthesis. Taking results of antibacterial activities of the 5 strains into consideration, strains s1b9-3, 10X7D-1-3, and s7b4-1 should be studied by order of importance to find potential antibacterial compounds.

## 5. Conclusion

In our study, the diversity, novelty, and antibacterial activity of cultivable actinobacteria from mangrove soil in Futian and Maoweihai of China were investigated. A total of 539 cultivable actinobacterial strains were identified and affiliated to 39 genera in 18 families of 8 orders. Eleven strains were considered as potential new taxa. The antibacterial assays showed 54 strains in 23 genera had antagonistic activities against at least one of “ESKAPE” bacteria, and the screening results based on pDualrep2 reporter system indicated the cultural broth of 5 strains could cause ribosome stalling as erythromycin did. Comprehensive analyses of all results in present study reveal that* streptomycetes* and rare actinobacteria isolated from mangrove soil are valuable sources to find new antibiotics. Notably, it seems that culture broths of* streptomyces *more frequently exhibit inhibitory activities against Gram-positive bacteria such as* E*.* faecium* and* S*.* aureus* than against Gram-negative bacteria such as* P*.* aeruginosa*. Sensitive and reliable screening model based on mechanism of action can accelerate the selection of target strains for further chemical studies.

## Figures and Tables

**Table 1:** Information of soil samples.

Samples	Sampling sites	Location	The characteristic of soil	Sampling depth
Sample 1	Futian	22°31'45.82” N 114°00'09.04” E	Rhizosphere soil of *Aegiceras corniculatum*	5 cm under surface
Sample 2	Futian	22°31'45.79” N 114°00'09.00” E	Rhizosphere soil of *Aegiceras corniculatum*	5 cm under surface
Sample 3	Maoweihai	21°51'20.51” N 108°36'14.11” E	Muddy soil	10 cm under surface
Sample 4	Maoweihai	21°51'20.58” N 108°36'14.12” E	Rhizosphere soil of *Aegiceras corniculatum*	10 cm under surface
Sample 5	Maoweihai	21°44'35.73” N 108°35'40.85” E	Rhizosphere soil of *Aegiceras corniculatum*	10 cm under surface
Sample 6	Maoweihai	21°44'35.84” N 108°35'40.87” E	Muddy soil	10 cm under surface
Sample 7	Maoweihai	21°44'36.30” N 108°35'40.93” E	Muddy soil	10 cm under surface
Sample 8	Maoweihai	21°44'36.44” N 108°35'40.82” E	Muddy soil	10 cm under surface
Sample 9	Maoweihai	21°44'36.03” N 108°35'40.69” E	Muddy soil	10 cm under surface
Sample 10	Maoweihai	21°44'36.10” N 108°35'40.50” E	Muddy soil	10 cm under surface

**Table 2:** Information on genera distribution of actinobacterial strains.

Genera	No. of isolates	No. of strains for assay	No. of strains with antibacterial activity
*Streptomyces*	86	27	20
*Microbacterium*	78	6	0
*Agromyces*	77	5	2
*Rhodococcus*	64	6	4
*Sinomonas*	46	5	3
*Mycobacterium*	28	3	0
*Curtobacterium*	23	2	0
*Nocardia*	20	7	2
*Arthrobacter*	15	3	1
*Leifsonia*	13	4	1
*Paenarthrobacter*	11	1	1
*Kocuria*	10	5	1
*Nocardiopsis*	7	4	3
*Brachybacterium*	7	1	0
*Agrococcus*	6	2	0
*Glutamicibacter*	6	2	1
*Kitasatospora*	5	1	1
*Isoptericola*	4	1	1
*Mycolicibacterium*	4	4	2
*Aeromicrobium*	3	1	0
*Brevibacterium*	2	1	0
*Schumannella*	2	2	0
*Micrococcus*	2	2	1
*Arsenicicoccus*	2	2	0
*Cellulosimicrobium*	2	2	1
*Gordonia*	2	2	2
*Micromonospora*	2	2	2
*Pseudarthrobacter*	1	1	1
*Homoserinibacter*	1	1	1
*Amnibacterium*	1	1	1
*Frigoribacterium*	1	1	0
*Oerskovia*	1	1	0
*Janibacter*	1	1	0
*Streptosporangium*	1	1	0
*Actinomadura*	1	1	1
*Modestobacter*	1	1	0
*Pseudonocardia*	1	1	1
*Nocardioides*	1	1	0
*Microlunatus*	1	1	0
Total number	539	115	54

**Table 3:** The sequence analyses based on almost full-length 16S rRNA gene (> 1321 bp) of 11 potential new species.

Strain	Accession number	Closest type species	Similarity of 16S rRNA gene sequence
9X7D-4	MK589797	*Agromyces brachium* IFO 16238^T^	98.1 %
2X8D-4	MK589796	*Agromyces neolithicus* 23-23^T^	98.1 %
9X9A-10	MK589795	*Agromyces luteolus* IFO 16235^T^	98.4 %
s7b8-3	MK589792	*Agromyces italicus* DSM 16388^T^	98.2 %
s6c9-2a	MK589794	*Agromyces binzhouensis* OAct353^T^	98.3 %
s6c8-3a	MK589793	*Agromyces binzhouensis* OAct353^T^	98.4 %,
7X8A-10	MK589799	*Agromyces tropicus* CM9-9^T^	98.3 %
7X7D-2	MK589798	*Agromyces tropicus* CM9-9^T^	97.2 %
10F1B-8-1	MK589789	*Homoserinibacter gongjuensis* 5GH26-15^T^	97.7 %
10F1B-5-1	MK589790	*Schumannella luteola* KHIA^T^	98.2 %
4F1A-5	MK589791	*Streptomyces deserti* C63^T^	98.0 %

## Data Availability

The data used to support the findings of this study are available from the corresponding authors upon request.

## Supplementary Materials

Supplementary Materials Table S1: Composition of ten different media used for isolation of actinobacteria from mangrove soil in Futian and Maoweihai. Table S2: Information on genera distribution of actinobacterial isolates from different samples. Table S3: Information on genera distribution of actinobacterial isolates recovered from the different culture media. Table S4: Antibacterial activity of 54 actinobacterial strains from mangrove soil in Futian and Maoweihai.
