# Supplementary material for: Studies on Antibacterial Activity and Diversity of Cultivable Actinobacteria Isolated from Mangrove Soil in Futian and Maoweihai of China
Source: Evid Based Complement Alternat Med. 2019 Jun 9;2019:3476567. doi: 10.1155/2019/3476567 (PMC6590530; doi:10.1155/2019/3476567)
Supplement: Supplementary Materials — Table S1: Composition of ten different media used for isolation of actinobacteria from mangrove soil in Futian and Maoweihai. Table S2: Information on genera distribution of actinobacterial isolates from different samples. Table S3: Information on genera distribution of actinobacterial isolates recovered from the different culture media. Table S4: Antibacterial activity of 54 actinobacterial strains from mangrove soil in Futian and Maoweihai. [file 3476567.f1.pdf]

Supplementary Materials

Table S1: Composition of ten different media used for isolation of actinobacteria from mangrove soil in Futian and Maoweihai.

Media	Composition (1 L distilled water)
M1	Starch 10.0 g, glucose 1.0 g, glycerinum 5.0 mL, trace salt solution 10.0 mL, agar 15.0 g, pH 6.0
M2	Glucose 4.0 g, yeast extract 4.0 g, malt extract 10.0 g, agar 15.0 g, pH 6.0
M3	Glucose 10.0 g, peptone 5.0 g, tryptone 3.0 g, NaCl 5.0 g, agar 15.0 g, pH 6.0
M4	Yeast extract 5.0 g, L-asparagine 1.0 g, glycerinum 10.0 g, K_2HPO_4 1.0 g, KNO_3 5.0 g, trace salt solution 1.0 mL, $MgCl_2$ 100.0 g, agar 15.0 g, pH 6.0
M5	Trehalose 5.0 g, proline 1.0 g, $(NH4)_2SO_4$ 1.0 g, NaCl 1.0 g, $CaCl_2$ 2.0 g, K_2HPO_4 1.0 g, $MgSO_4 \cdot 7H_2O$ 1.0 g, vitamin mixture 1.0 ml, agar 15.0 g, pH 6.0
M6	R2A agar (Difco), pH 6.0
M7	Glycerinum 15.0 g, L-tyrosine 0.5 g, L-asparagine 1.0 g, K_2HPO_4 0.5 g, $MgSO_4 \cdot 7H_2O$ 0.5 g, NaCl 0.5 g, $FeSO_4 \cdot 7H_2O$ 0.01 g, trace salt solution 1.0 ml, agar 15.0 g, pH 6.0
M8	Arginine 1.0 g, glycerinum 6.0 mL, vitamin mixture 1.0 ml, trace salt solution 1.0 ml, agar 15.0 g, pH 6.0
M9	Starch 10.0 g, casein 0.3 g, KNO_3 2.0 g, $MgSO_4 \cdot 7H_2O$ 0.05 g, NaCl 30.0 g, K_2HPO_4 2.0 g, $CaCO_3$ 0.02 g, $FeSO_4 \cdot 7H_2O$ 0.01 g, agar 15.0 g, pH 6.0
M10	L-Asparagine 1.0 g, glycerinum 10.0 g, K_2HPO_4 1.0 g, trace salt solution 1.0 ml, agar 15.0 g, pH 6.0

Note: Trace salt solution: $FeSO_4 \cdot 7H_2O$ 0.2 g, $MnCl_2.4H_2O$ 0.01 g, $ZnSO_4 \cdot 7H_2O$ 0.01 g, distilled water 100.0 ml.

Vitamin mixture: thiamine 0.1 g, pyridoxine 0.1 g, riboflavin 0.1g, niacin 0.1 g, biotin 0.1 g, distilled water 100.0 ml.

Table S2: Information on genera distribution of actinobacterial isolates from different samples.

Genera	Samples									
	S1	S2	S3	S4	S5	S6	S7	S8	S9	S10
Streptomyces	39	32	1	1	4	-	9	-	-	-
Kitasatospora	4	-	-	-	1	-	-	-	-	-
Agrococcus	2	-	-	1	-	1	-	-	2	-
Homoserinibacter	1	-	-	-	-	-	-	-	-	-
Agromyces	2	-	-	10	31	8	16	-	9	1
Curtobacterium	19	-	-	-	-	-	3	-	1	-
Glutamicibacter	5	-	-	-	1	-	-	-	-	-
Leifsonia	-	1	10	1	-	-	1	-	-	-
Microbacterium	29	3	2	1	13	6	10	-	9	5
Schumannella	2	-	-	-	-	-	-	-	-	-
Amnibacterium	-	-	1	-	-	-	-	-	-	-
Frigoribacterium	-	-	-	-	-	-	-	-	-	1
Oerskovia	-	1	-	-	-	-	-	-	-	-
Brachybacterium	-	4	-	1	-	2	-	-	-	-
Arthrobacter	-	11	-	-	2	1	1	-	-	-
Micrococcus	-	-	1	-	-	1	-	-	-	-
Paenarthrobacter	8	-	-	-	3	-	-	-	-	-
Kocuria	-	2	-	3	3	-	-	2	-	-
Sinomonas	-	2	16	16	6	-	2	1	3	-
Janibacter	-	-	-	-	1	-	-	-	-	-
Arsenicicoccus	-	1	-	-	-	1	-	-	-	-
Brevibacterium	2	-	-	-	-	-	-	-	-	-
Cellulosimicrobium	-	-	-	-	1	1	-	-	-	-
Isoptericola	1	-	-	-	3	-	-	-	-	-
Gordonia	-	-	-	-	-	-	1	-	1	-
Nocardia	5	5	-	3	-	-	5	-	2	-
Rhodococcus	14	12	2	1	9	1	11	-	11	3
Mycobacterium	2	8	-	2	3	2	8	-	3	-
Nocardiopsis	-	1	2	3	1	-	-	-	-	-
Streptosporangium	-	1	-	-	-	-	-	-	-	-
Actinomadura	-	-	-	-	-	1	-	-	-	-
Micromonospora	-	1	-	-	-	-	-	-	1	-
Modestobacter	-	-	-	-	1	-	-	-	-	-
Pseudonocardia	1	-	-	-	-	-	-	-	-	-
Nocardioides	-	1	-	-	-	-	-	-	-	-
Aeromicrobium	-	-	-	3	-	-	-	-	-	-
Microlunatus	-	1	-	-	-	-	-	-	-	-
Mycolicibacterium	2	1	-	-	-	-	-	-	1	-
Pseudarthrobacter	-	-	-	-	-	-	1	-	-	-
Total number of genera	17	18	8	13	16	11	12	2	11	4
Total number of isolates	138	88	35	46	83	25	68	3	43	10

Note: −, no isolate

Table S3: Information on genera distribution of actinobacterial isolates recovered from the different culture media.

Genera	Media									
	M1	M2	M3	M4	M5	M6	M7	M8	M9	M10
Streptomyces	2	-	-	7	10	8	17	18	10	14
Kitasatospora	-	-	-	-	-	1	1	3	-	-
Agrococcus	-	-	2	1	-	-	1	1	-	1
Homoserinibacter	-	-	-	-	-	-	-	-	-	1
Agromyces	-	4	6	2	-	-	24	5	34	2
Curtobacterium	-	-	3	-	1	6	6	-	5	2
Glutamicibacter	-	-	1	-	-	1	1	-	2	1
Leifsonia	-	2	1	-	-	3	3	2	-	2
Microbacterium	-	2	10	14	1	17	12	8	10	4
Schumannella	-	-	-	-	-	-	-	-	-	2
Amnibacterium	-	-	-	-	-	1	-	-	-	-
Frigoribacterium	-	-	1	-	-	-	-	-	-	-
Oerskovia	-	-	-	-	-	-	1	-	-	-
Brachybacterium	3	1	1	-	-	-	1	-	-	1
Arthrobacter	-	1	3	1	1	2	2	1	3	1
Micrococcus	-	-	1	-	-	-	-	-	1	-
Paenarthrobacter	-	-	3	-	-	2	2	-	2	2
Kocuria	-	-	1	2	-	1	3	2	1	-
Sinomonas	3	2	6	-	5	5	4	4	9	8
Janibacter	-	-	-	-	-	-	-	-	1	-
Arsenicicoccus	-	-	-	-	1	-	-	-	-	1
Brevibacterium	-	-	-	-	-	2	-	-	-	-
Cellulosimicrobium	-	-	-	1	-	-	1	-	-	-
Isoptericola	-	-	-	3	-	-	1	-	-	-
Gordonia	-	-	1	1	-	-	-	-	-	-
Nocardia	-	3	-	1	-	1	7	4	2	2
Rhodococcus	2	3	13	18	-	4	12	5	2	5
Mycobacterium	-	4	2	-	-	5	4	3	1	9
Nocardiopsis	-	-	-	2	-	-	1	2	2	-
Streptosporangium	-	-	-	-	1	-	-	-	-	-
Actinomadura	-	-	-	-	-	1	-	-	-	-
Micromonospora	-	-	-	-	1	-	1	-	-	-
Modestobacter	-	-	-	-	-	1	-	-	-	-
Pseudonocardia	-	-	-	1	-	-	-	-	-	-
Nocardioides	-	-	-	-	1	-	-	-	-	-
Aeromicrobium	-	-	-	3	-	-	-	-	-	-
Microlunatus	-	-	-	-	-	-	1	-	-	-
Mycolicibacterium	-	-	-	-	-	-	1	1	1	1
Pseudarthrobacter	-	1	-	-	-	-	-	-	-	-
Total number of genera	4	10	16	14	9	17	23	14	16	18
Total number of isolates	10	23	55	57	22	61	107	59	86	59

Note: −, no isolate.

Table S4: Antibacterial activity of 54 actinobacterial strains from mangrove soil in Futian and Maoweihai.

Isolates (GenBank accession no.)	Closet type strain (Similarity, %)	Fractions*	Indicator bacteria											
			E. coli		*P. aeruginosa*		*K. peneumoniae*		*A. baumannii*		*E. faecalis*		*S. aureus*	
			S	R	S	R	S	R	S	R	S	R	S	R
4F1A-3-1 (MK589748)	*Streptomyces antibioticus* NBRC 12838^T (100 %)	E	-	-	-	-	-	-	8.3	-	11.6	21.8	17.3	20.2
		W	-	-	-	-	-	-	-	-	8.8	14.2	11.4	9.4
		M	-	-	-	-	-	-	-	-	16.0	21.4	17.2	16.0
8J6B-1 (MK589749)	*Streptomyces jiujiangensis* JXJ 0074^T (99.4 %)	E	-	-	-	-	18.0	-	9.5	9.2	-	-	15.1	-
		W	-	-	-	-	-	-	-	-	-	-	-	-
		M	-	-	-	-	-	-	-	-	-	-	-	-
4F1B-7 (MK589747)	*Streptomyces antibioticus* NBRC 12838^T (100 %)	E	-	-	-	-	-	-	-	-	13.4	19.0	16.2	20.4
		W	-	-	-	-	-	-	-	8.5	7.5	15.0	12.0	13.1
		M	-	-	-	-	-	-	10.2	13.8	18.3	22.9	18.0	18.9
9F1A-6 (MK589746)	*Streptomyces zaomyceticus* NBRC 13348^T (100 %)	E	-	-	-	-	-	-	-	-	-	-	-	-
		W	-	-	-	-	-	-	-	-	-	-	-	-
		M	-	-	-	-	-	-	-	-	-	-	-	23.1
4F1A-4 (MK589745)	*Streptomyces parvulus* NBRC 13193^T (100 %)	E	-	-	-	-	-	-	9.9	9.9	14.3	24.5	22.0	-
		W	-	-	-	-	-	-	-	-	-	18.3	12.9	13.6
		M	-	-	-	-	-	-	9.6	9.4	20.5	26.8	23.0	18.8
5F1A-4 (MK589744)	*Streptomyces antibioticus* NBRC 12838^T (100 %)	E	-	-	-	-	-	-	-	-	11.5	17.7	16.7	19.3
		W	-	-	-	-	-	-	-	-	9.4	11.9	8.4	9.1
		M	-	-	-	-	-	-	-	-	14.7	22.7	16.4	12.8
10X7D-1-3 (MK589743)	*Streptomyces angustmyceticus* NRRL B-2347^T (99.9 %)	E	-	-	-	-	-	-	-	-	-	9.8	14.5	-
		W	9.8	-	-	-	10.7	-	-	-	10.0	-	14.9	10.7

		M	-	-	-	-	10.0	-	-	-	8.3	-	8.8	10.0
7X8A-5	*Streptomyces wuyuanensis* CGMCC 4.7042^T	E	-	-	-	-	-	-	-	-	-	7.0	-	-
(MK589742)	(100 %)	W	-	-	-	-	-	-	-	-	-	11.6	-	-
		M	-	-	-	-	-	-	-	-	-	-	-	-
7X8A-13	*Streptomyces chattanoogensis* NRRL ISP-5002^T	E	19.4	18.8	-	-	-	8.6	9.6	-	-	10.6	22.2	16.3
(MK589741)	(99.9 %)	W	-	9.3	-	-	-	-	-	-	-	13.1	-	-
		M	14.9	10.6	-	-	11.8	16.0	-	-	-	9.1	-	-
10F1D-1F	*Streptomyces pratensis* ch24^T (100 %)	E	-	-	-	-	-	-	-	-	-	-	-	-
(MK589740)		W	-	-	-	-	-	-	-	-	-	-	-	-
		M	-	-	-	-	-	-	-	-	-	-	8.4	-
4F1A-5	*Streptomyces deserti* C63^T (98.0 %)	E	-	-	-	-	-	-	-	-	-	-	-	-
(MK589791)		W	-	-	-	-	-	-	-	-	-	-	-	-
		M	-	-	-	-	-	-	-	-	8.0	9.0	11.3	-
10F1D-3	*Streptomyces virginiae* NRRL ISP-5094^T (100 %)	E	-	-	-	-	-	-	-	-	-	-	-	-
(MK589738)		W	15.2	16.4	-	-	14.3	13.4	10.6	10.9	-	9.7	-	-
		M	17.7	21.3	-	-	16.8	18.1	12.4	8.6	9.4	-	-	-
7F1D-2	*Streptomyces zaomyceticus* NBRC 13348^T (99.9 %)	E	-	-	-	-	-	-	-	-	-	-	-	-
(MK589737)		W	-	-	-	-	-	-	-	-	-	-	-	-
		M	-	-	-	-	-	-	-	-	-	9.0	-	-
10F1B-6	*Streptomyces amritsarensis* MTCC 11845^T (100 %)	E	-	-	-	-	-	-	-	-	-	-	-	-
(MK589736)		W	-	-	-	-	-	-	-	-	-	-	-	-
		M	-	-	-	-	-	-	-	-	8.8	9.0	-	-
7X8A-6	*Streptomyces sanglieri* NBRC 100784^T (99.6 %)	E	-	-	-	-	-	-	-	-	-	-	-	12.0
(MK589735)		W	-	-	-	-	-	-	-	-	-	-	-	-
		M	-	-	-	-	-	-	-	-	-	-	-	-
10F1A-2	*Streptomyces drozdowiczii* NBRC 101007^T (99.4 %)	E	-	-	-	-	-	-	-	-	-	-	12.8	-

(MK589734)		W	-	-	-	-	-	-	-	-	-	-	11.2	16.3
		M	-	-	-	-	-	-	-	-	-	-	-	-
5X8A-1	*Streptomyces samsunensis* M1463^{T} (100 %)	E	9.1	-	-	-	-	-	-	-	17.6	19.6	29.0	27.8
(MK589733)		W	-	-	-	-	-	-	-	-	11.7	12.8	-	-
		M	-	-	-	-	-	-	-	-	20.8	23.8	17.0	13.6
7X7B-4	*Streptomyces roseolus* NBRC 12816^{T} (99.8 %)	E	-	-	-	-	-	-	-	-	8.9	12.0	17.3	21.6
(MK589732)		W	-	-	-	-	-	-	-	-	-	-	-	-
		M	-	-	-	-	-	-	-	-	-	-	-	-
7F1A-7F	*Streptomyces angustmyceticus* NRRL B-2347^{T}	E	-	-	-	-	-	-	-	-	8.8	12.2	16.0	12.3
(MK589739)	(100 %)	W	-	-	-	-	-	-	-	-	-	10.6	12.9	12.0
		M	-	-	-	-	-	-	-	-	-	-	-	-
s1b9-3	*Streptomyces angustmyceticus* NRRL B-2347^{T}	E	13.0	10.7	-	-	13.7	-	8.4	-	9.6	-	18.6	-
(MK589727)	(99.8 %)	W	-	13.5	-	-	13.3	-	12.5	12.8	14.0	-	-	8.3
		M	-	-	-	-	8.2	-	-	-	-	-	8.2	8.9
4F1B-1-2	*Rhodococcus ruber* DSM 43338^{T} (99.9 %)	E	-	-	-	-	-	-	-	-	-	-	-	-
(MK685120)		W	10.6	-	-	-	-	-	-	-	-	-	-	-
		M	-	-	-	-	-	-	-	-	-	-	-	-
3X9B-4	*Rhodococcus gordoniae* DSM 44689^{T} (100 %)	E	-	-	-	-	-	-	-	-	-	-	-	-
(MK589750)		W	13.1	-	-	-	-	-	-	-	-	-	-	-
		M	-	-	-	-	-	-	-	-	-	-	-	-
3X7A-10	*Rhodococcus pedocola* UC12^{T} (99.9 %)	E	-	-	-	-	-	-	-	-	-	-	-	-
(MK589771)		W	14.0	-	-	-	-	-	-	-	-	-	-	-
		M	-	-	-	-	-	-	-	-	-	-	-	-
s1c7-14	*Rhodococcus defluvii* Ca11^{T} (99.8 %)	E	-	-	-	-	-	-	-	-	-	-	9.7	-
(MK589729)		W	-	-	-	-	-	-	-	-	-	-	-	-
		M	-	-	-	-	-	-	-	-	-	-	-	-

9X8B-1	*Sinomonas atrocyanea* KCTC 3377^T (98.9 %)	E	-	-	-	-	-	-	-	-	-	-	-	-
(MK589770)		W	-	-	-	-	-	-	-	-	-	-	-	-
		M	-	-	-	-	9.3	-	-	-	-	-	-	18.8
6X7D-4	*Sinomonas flava* CW 108^T (99.6 %)	E	-	-	-	-	-	-	-	-	-	-	-	-
(MK589754)		W	-	-	-	-	-	-	-	-	-	-	-	-
		M	-	-	-	-	9.7	-	-	-	-	-	-	-
s6b7-4	*Sinomonas soli* CW 59^T (99.6 %)	E	-	-	-	-	-	-	-	-	-	-	10.2	-
(MK589725)		W	-	-	-	-	-	-	-	-	-	-	-	-
		M	-	-	-	-	-	-	-	-	-	-	-	-
4X7A-3	*Nocardiopsis alba* DSM 43377^T (99.2 %)	E	-	-	-	-	-	-	9.0	-	-	11.0	14.6	19.6
(MK589731)		W	-	-	-	-	-	-	-	-	-	-	-	-
		M	-	-	-	-	-	-	-	-	8.4	-	-	-
9J6A-2	*Nocardiopsis synnemataformans* DSM 44143^T	E	-	-	-	-	-	-	-	-	-	-	19.0	15.9
(MK589773)	(99.4 %)	W	-	-	-	-	-	-	-	-	-	20.9	-	-
		M	-	-	-	-	-	-	-	-	11.6	16.3	-	-
9J6A-1	*Nocardiopsis synnemataformans* DSM 44143^T	E	-	-	-	-	-	-	-	-	-	-	-	-
(MK589753)	(99.3 %)	W	8.9	-	-	-	-	-	-	-	-	-	-	-
		M	-	-	-	-	-	-	-	-	-	-	-	-
7X8D-2	*Agromyces aurantiacus* YIM 21741^T (98.2 %)	E	-	-	-	-	-	-	-	-	-	-	-	-
(MK589755)		W	-	-	-	-	-	-	-	-	-	-	-	-
		M	-	-	-	-	-	-	-	-	-	-	-	8.0
9X8D-2	*Agromyces binzhouensis* OAct353^T (99.8 %)	E	-	-	-	-	-	-	-	-	-	-	-	-
(MK589763)		W	8.8	-	-	-	-	-	-	-	-	-	-	-
		M	-	-	-	-	-	-	-	-	-	-	-	-
s1b4-6	*Nocardia shimofusensis* NBRC 100134^T (100 %)	E	-	-	-	-	-	-	-	-	-	-	-	-
(MK589728)		W	-	-	-	9.7	-	-	-	-	-	-	-	-

		M	-	-	-	-	-	-	-	-	-	-	-	-
7F1A-7	*Nocardia asteroides* NBRC 15531^T (99.6 %)	E	-	-	-	-	-	-	-	-	-	-	-	-
(MK589739)		W	8.9	-	-	-	-	-	-	-	-	-	-	-
		M	-	-	-	-	-	-	-	-	-	-	-	-
8F1A-1-1	*Mycolicibacterium fluoranthenivorans* FA-4^T	E	-	-	-	-	-	-	-	-	-	-	-	-
(MK589751)	(98.9 %)	W	9.0	-	-	-	-	-	-	-	-	-	-	-
		M	-	-	-	-	-	-	-	-	-	-	-	-
10F1D-2	*Mycolicibacterium anyangense* QIA-38^T (99.2 %)	E	-	-	-	-	-	-	-	-	-	-	-	-
(MK589788)		W	-	-	-	-	-	-	-	-	-	14.0	-	-
		M	-	-	-	-	-	-	-	-	-	-	-	-
4X9B-1	*Gordonia westfalica* DSM 44215^T (99.8 %)	E	-	-	-	-	-	-	-	-	-	-	-	-
(MK589752)		W	-	11.4	-	-	11.4	-	-	-	-	-	-	-
		M	12.3	14.8	-	-	9.1	-	-	-	-	-	-	-
3X8D-4	*Gordonia bronchialis* DSM 43247^T (100 %)	E	-	-	-	-	-	-	-	-	-	-	-	-
(MK589786)		W	-	9.1	-	-	12.7	-	-	-	-	-	-	-
		M	11.4	12.3	-	-	-	-	-	-	-	-	-	-
s1d5-4	*Micromonospora carbonacea* DSM 43168^T (100 %)	E	-	-	-	-	8.6	-	-	-	-	-	10.8	-
(MK589730)		W	-	-	-	-	-	-	-	-	-	-	-	-
		M	-	-	-	-	-	-	-	-	-	-	-	-
7X9A-12	*Micromonospora citrea* DSM 43903^T (99.6 %)	E	-	-	-	-	-	-	-	-	-	-	-	-
(MK589777)		W	16.8	11.3	-	-	-	-	-	-	-	-	-	-
		M	-	-	-	-	-	-	-	-	-	-	-	-
6X7D-2-2	*Arthrobacter agilis* KCTC 3200^T (98.7 %)	E	-	-	-	-	-	-	-	-	-	-	-	-
(MK589756)		W	10.1	-	-	-	-	-	-	-	-	-	-	-
		M	-	-	-	-	-	-	-	-	-	-	-	-
s6b7-3a	*Leifsonia shinshuensis* JCM 10591^T (99.5 %)	E	-	-	-	-	8.2	-	-	-	-	-	20.3	21.2

(MK589762)		W	-	8.9	-	-	-	-	-	-	-	-	-	-
		M	-	-	-	-	-	-	-	-	-	-	-	-
6F1D-2	*Paenarthrobacter ureafaciens* DSM 20126^T	E	-	-	-	-	-	-	-	-	-	9.4	-	-
(MK589766)	(99.8 %)	W	-	-	-	-	-	-	-	-	-	-	-	-
		M	-	-	-	-	-	-	-	-	-	-	-	-
s1c8-3b	*Kocuria polaris* CMS 76or^T (100 %)	E	-	-	-	-	-	-	-	-	-	-	-	-
(MK589726)		W	-	-	-	9.8	-	-	-	-	-	-	-	-
		M	-	-	-	-	-	-	-	-	-	-	-	-
7F1D-1	*Glutamicibacter arilaitensis* Re117^T (99.2%)	E	-	-	-	-	-	-	-	-	-	-	8.0	-
(MK589767)		W	-	-	-	-	-	-	-	-	-	-	-	-
		M	-	-	-	-	-	-	-	-	-	-	-	-
6F1D-1	*Kitasatospora psammotica* NBRC 13971^T (100 %)	E	-	-	-	-	-	-	-	-	-	-	-	-
(MK589787)		W	-	-	-	-	-	-	-	-	-	-	12.7	-
		M	-	-	-	-	-	-	-	-	-	-	-	-
4X7D-1	*Isoptericola jiangsuensis* CLG^T (99.72 %)	E	-	-	-	-	-	-	-	-	-	-	-	-
(MK589781)		W	18.4	9.9	-	-	-	-	-	-	-	-	-	-
		M	-	-	-	-	-	-	-	-	-	-	-	-
3J6B-3	*Micrococcus aloeverae* AE-6^T (100 %)	E	-	-	-	-	-	-	-	-	-	-	-	-
(MK589759)		W	10.6	-	-	-	-	-	-	-	-	-	-	-
		M	-	-	-	-	-	-	-	-	-	-	-	-
s7b4-1	*Cellulosimicrobium cellulans* LMG 16121^T (99.0 %)	E	-	-	-	-	-	-	-	-	-	-	7.0	-
(MK589760)		W	-	-	-	-	-	-	-	-	-	-	-	8.0
		M	-	-	-	-	-	-	-	-	-	-	-	-
2X8D-3	*Pseudarthrobacter niigatensis* LC4^T (100 %)	E	-	-	-	-	-	-	-	-	-	-	-	-
(MK589769)		W	-	-	-	-	-	-	-	-	-	-	-	-
		M	-	-	-	-	-	-	-	-	-	-	12.6	14.8

10F1B-8-1	*Homoserinibacter gongjuensis* 5GH26-15^{T} (97.7 %)	E	-	-	-	-	-	-	-	-	-	-	-	-
(MK589789)		W	9.4	10.6	-	-	-	-	-	-	-	-	-	18.8
		M	-	-	-	-	-	-	-	-	-	-	-	-
6J6B-5	*Amnibacterium kyonggiense* KSL51201-037^{T}	E	-	-	-	-	-	-	-	-	-	-	9.4	-
(MK589780)	(99.4 %)	W	8.4	-	-	-	-	-	-	-	-	-	-	-
		M	-	-	-	-	-	-	-	-	-	-	-	-
s7c6-2a	*Actinomadura maheshkhaliensis* 13-12-50^{T} (100 %)	E	-	-	-	-	-	-	-	-	-	-	13.3	-
(MK589776)		W	-	-	-	-	-	-	-	-	-	-	-	-
		M	-	-	-	-	-	-	-	-	-	-	-	-
4F1A-3	*Pseudonocardia carboxydivorans* Y8^{T} (100 %)	E	-	-	-	-	-	-	-	-	-	-	9.5	9.8
(MK589774)		W	11.7	-	-	-	8.5	-	-	-	-	-	10.2	-
		M	-	-	-	-	-	-	-	-	-	-	-	-
		methanol	-	-	-	-	-	-	-	-	-	-	-	-
		levofloxacin	26.1	24.5	12.8	9.8	26.8	14.0	19.0	15.0	12.6	10.4	19.3	15.5

Note: Diameter of the inhibitory zone: mm; −, no inhibitory activity.

* E: Sample from ethyl acetate layer; W: Sample from water layer; M: Sample from mycelium.

S: Sensitive strains; R: Drug-resistant strains.
